# Individuals in the prediabetes stage exhibit reduced hippocampal tail volume and executive dysfunction

**DOI:** 10.1002/brb3.1351

**Published:** 2019-06-25

**Authors:** Sun Dong, Lu Dongwei, Junjian Zhang, Jinyu Liang, Zhenmeng Sun, Jian Fang

**Affiliations:** ^1^ Department of Neurology Zhongnan Hospital of Wuhan University Wuhan China; ^2^ Department of Radiology Zhongnan Hospital of Wuhan University Wuhan China

**Keywords:** cognitive impairment, diabetes, hippocampal subfield volume, prediabetes

## Abstract

**Introduction:**

High glucose levels are associated with cognitive impairment and total hippocampal volume reductions. However, the effects of the blood glucose level on hippocampal subfield volumes remain unclear, especially in the prediabetes stage.

**Methods:**

Sixty participants were enrolled in this cross‐sectional study and were divided into the nondiabetes, prediabetes, and diabetes groups according to their medical history and A1c level. A full battery of neuropsychological tests was used to assess the global cognition, executive function, attention, verbal fluency, working memory, immediate memory, and delayed memory. FreeSurfer 6.0 was used for the hippocampus parcellation. Hippocampal subfield volumes were adjusted by intracranial volume. Analyses of covariance, multiple linear regression, and partial correlation analysis were used to explore the relationship between A1c level, cognitive function, and hippocampal subfields volume, in which age, sex, education years, body mass index, history of hypertension, level of cholesterol, and the presence of ApoE4‐positive status were adjusted.

**Results:**

Significant differences in the total left hippocampal volume (*p* = 0.046) and left hippocampal tail volume (*p* = 0.014) were noted among three groups. Significant correlation was identified between the A1c level and the volume of left hippocampal tail (*r* = −0.334, *p* = 0.009) after adjusting all the covariants. Increased A1c level was significantly associated with executive dysfunction, as assessed by trail making test B (*R* = 0.503, *p* = 0.0016) and Stroop test C (*R* = 0.506, *p* = 0.001).

**Conclusions:**

Our results support that the left hippocampal tail volume may be served as an early marker of diabetes‐related brain damage, associated with executive dysfunction. Clinicians should pay closer attention to adults in the prediabetes stage to prevent later cognitive impairment.

## 1 INTRODUCTION

Diabetes in the midlife was shown to increase the risk of cognitive impairment and dementia in the late life, wherein the hippocampal atrophy was one of the important mechanisms Biessels, Staekenborg, Brunner, Brayne, & Scheltens, 2006; Moran et al., 2013; Roberts et al., 2014). Even in individuals without diabetes, higher glucose level is associated with cognitive impairment and the loss of hippocampal volume (Cherbuin, Sachdev, & Anstey, 2012; Crane et al., 2013). These findings suggest that the hippocampus is particularly vulnerable to hyperglycemia. However, the hippocampal formation is a heterogeneous structure, consisting of several histologically distinguishable modules, such as the cornu ammonis (CA) regions, dentate gyrus (DG), subiculum, and presubiculum. Each region is associated with differential functions in various diseases (Bartsch, Dohring, Rohr, Jansen, & Deuschl, 2011; Pievani et al., 2011). Therefore, further research would be required to elaborate the effect of hyperglycemia on hippocampal subdivision.

Currently, it still remains elusive for the association of the plasma blood glucose levels and the hippocampal subfield volumes, especially in the prediabetes stage. One study revealed that diabetes was associated with global hippocampal atrophy, driven by dysfunction of DG (Wu et al., 2008). However, this study included participants of up to 80 years of age and most of them had previous cerebral infarction, two factors (age and ischemic stroke) which have likewise been linked to hippocampal atrophy. Another study showed that the loss of subiculum and CA1 volumes was more pronounced in patients with diabetes than the controls (Zhang et al., 2015). Nevertheless, these studies did not distinguish those with prediabetes from the normal controls, thus making it still difficult to know which hippocampal subfield is affected in the early stage of diabetes.

In the present study, we used FreeSurfer version 6.0 to precisely segment the bilateral hippocampus and then explored the relationship between the plasma glycosylated hemoglobin (A1c) level and the hippocampal subfield volumes and cognitive performance in a relatively younger population.

## 2 METHODS

### 2.1 Participants

In total, 60 volunteers (30 men and 30 women; mean age, 58.4 ± 4.9 years) from the memory clinic of Zhongnan Hospital of Wuhan University were enrolled in this study. Inclusion criteria were as follows: (a) from 50 to 70 years; (b) dementia‐free (assessed by DSM‐5) and stroke‐free (assessed by medical history and brain MR imaging); and (c) education level higher than middle school. Exclusion criteria were as follows: (a) participants with contraindications to MR imaging; (b) other central nervous system diseases (such as intracranial infection, demyelinating diseases, or brain tumor); (c) severe depressive or anxious status assessed by medical history and Hamilton Depression and Hamilton Anxiety Scales; and (d) patients with hypoglycemic manifestations in the past 1 year (hunger, panic, cold sweat, and random blood sugar < 3.9 mmol/L). All the subjects underwent physical examinations, vascular risk factor evaluation, a series of complete neuropsychological assessments, and magnetic resonance imaging (MRI) scanning. This study was approved by the medical ethics committee of Zhongnan Hospital, Wuhan University. Written informed consent was obtained from each participant (clinical research registration number: chiCTR‐RNC‐12002205).

### 2.2 Vascular risk factors assessment

Vascular risk factors (VRFs) were determined based on the participants’ medical history and clinical examinations. Diabetes mellitus, hypertension, current smoking status, higher body mass index (BMI), serum lipid level, physical activity, and presence of ApoE4 status were recorded in details. The mean arterial pressure was calculated according to the results of 24‐hr ambulatory blood pressure monitoring. The plasma A1c level (obtained by Beckman Synchron System) was used to assess the average blood glucose level. All participants were divided into three groups according to their medical history and the A1c level, with the criteria as follows (American Diabetes Association, 2018): nondiabetes group, A1c < 5.7%; prediabetes group, 5.7% ≤ A1c < 6.5%; and type 2 diabetes group, A1c ≥ 6.5%.

### 2.3 Neuropsychological assessments

All participants underwent a full battery of neuropsychological assessments, which included global cognitive function (Montreal Cognitive Assessment, MoCA [Nasreddine et al., 2005]), immediate and delayed memory (Rey auditory verbal learning test [Elst, Boxtel, Breukelen, & Jolles, 2005]), executive function (Stroop color and word tests [Lee & Chan, 2000]), and verbal fluency (verbal fluency test [Mok, Lam, & Chiu, 2004]), and executive function with visuomotor tracking and attention ability (trail making test part A and part B [Lu & Bigler, 2002]). The Hamilton Anxiety Scale and Hamilton Depression Scale (Leung, Wing, Kwong, Lo, & Shum, 1999) were used to exclude those with severe anxiety or severe depression. All the tests were assessed by trained and experienced neurologists.

### 2.4 MR imaging

The MRI sequences include the three‐dimensional T1‐weighted and T2 FLAIR sequence. We obtained T1‐weighted images using a single 3‐Tesla MR scanner (MAGNETOM Trio, Siemens). Magnetization‐prepared rapid gradient‐echo imaging was conducted to acquire high‐resolution three‐dimensional T1‐weighted images according to the following protocol: repetition time = 1900 ms, echo time = 1.92 ms, inversion time = 900 ms, flip angle = 9°, thickness = 1.0 mm, field of view = 256 mm × 256 mm, and voxel size: 1.0 × 1.0 × 1.0 mm^3^. A total of 176 images were collected sagittally from the whole brain. T2 fluid attenuated inversion recovery (FLAIR) sequence was obtained using the following parameters: repetition time = 7,000 ms, echo time = 94 ms, inversion time = 2,210 ms, flip angle = 130°, thickness = 6.0 mm, spacing between slices = 7.8 mm. Number of lacunes and Fazekas classification were evaluated by 2 trained radiologists.

### 2.5 MR image processing

Volumetric analyses were performed on the three‐dimensional T1‐weighted magnetization‐prepared rapid gradient‐echo images. After transforming the raw data into the Nifti format using MRIcron (https://www.nitrc.org/projects/mricron), hippocampal subfield segmentation was performed using the FreeSurfer image analysis software version 6.0 (Fischl & Dale, 2000), which is documented and freely available for download online. Hippocampal subfields were divided as follows: CA1, CA2/3, CA4, fimbria, DG, hippocampal–amygdaloid transition region, hippocampal tail, hippocampal fissure, molecular layer, parasubiculum, presubiculum, and subiculum. The total intracranial volume (ICV) was calculated on the T1‐weighted images using SPM12 (Malone et al., 2015). The global hippocampal volume was adjusted for the ICV using the following covariance formula:

$$\begin{array}{c} \text{Adjusted hippocampal volume}=\text{raw hippocampal volume}\\ -b\times (\text{ICV}-\text{mean ICV}) \end{array}$$

where *b* is the slope of a regression of a region‐of‐interest volume of the ICV (Buckner et al., 2004). We take left hippocampal volume for example (a) make the linear regression between ICV and hippocampal volume, wherein hippocampal volume is the dependent variable and ICV is the independent variable; (b) *b* is the slope of the regression; (c) calculate the ICV‐mean ICV, followed by multiplying the respective *b* value; (d) finish the rest according to the formula as follows: HCV_adj_ = HCV_nat_−*b*(ICV−mean ICV_nat_); and (e) adjust each subfield hippocampal volume according to this way. This approach yields a distribution that is more Gaussian than the distribution obtained using a ratio approach.

### 2.6 Statistical analysis

We used SPSS 19.0 (SPSS Science Inc.) and Prism 5 (GraphPad Software) to analyze the data. Normality was tested using the Shapiro–Wilk test. Group comparisons of clinical and demographic data were conducted using analysis of variance (ANOVA) for continuous variables and chi‐squared tests for categorical variables. Analysis of covariance (ANCOVA) was used to compare group differences for cognitive performance and hippocampal subfields, wherein age, gender, education years were adjusted in model 1, followed by additional adjustment for BMI, hypertension, cholesterol level, presence of ApoE4‐positive status in model 2. We analyzed the interaction between A1c categories and age in ANCOVA. Sidak analysis was used for post hoc analysis. Multiple linear regression with enter method was performed for cognitive function comparison, with age, gender, education years, BMI, history of hypertension, cholesterol level, and presence of ApoE4‐positive status in the model. Partial correlation analysis was used to correlate the A1c value with the hippocampal subfields volume and cognitive performance, in which age, gender, education years, BMI, history of hypertension, cholesterol level, and presence of ApoE4‐positive status as covariants. It was considered statistically significant when the *p*‐value was <0.05 two‐sided.

## 3 RESULTS

### 3.1 Study population

Table 1 presents the demographic and clinical data for all participants. No significant differences were identified among groups for age, sex distribution, duration of education, presence of ApoE4, or other main vascular risk factors. No significant differences for the CSF, ICV, gray matter volume, and white matter volume were noted among the groups. In particular, the presence of lacunes and WMHs was not significantly different among groups. Only 2 subjects had lacunae, and only 1 subject had severe WMHs (Fazekas score ≥ 2).

**Table 1:** Between‐group differences in demographic and clinical characteristics, as evaluated by analyses of variance or chi‐squared tests

	Nondiabetes *n* = 22	Prediabetes *n* = 17	Diabetes *n* = 21	*p*‐Value
Age, years	57.2 ± 4.1	58.1 ± 4.6	59.3 ± 5.1	0.347
Sex (female, %)	10 (45.5%)	9 (52.9%)	11 (52.4%)	0.898
Education, years	13.5 ± 2.8	14.4 ± 1.7	14.9 ± 1.9	0.297
History of hypertension (%)	14 (63.6%)	13 (76.5%)	12 (57.1%)	0.394
SBP, mmHg	129.1 ± 13.1	134.8 ± 12.9	130.4 ± 13.8	0.282
DBP, mmHg	77.8 ± 10.4	80.1 ± 11.6	79.4 ± 10.8	0.419
History of hyperlipidemia (%)	13 (59.1%)	12 (70.6%)	15 (71.4%)	0.244
Total cholesterol, mmol/L	4.8 ± 1.0	4.7 ± 0.9	4.9 ± 0.7	0.916
BMI, kg/m^2^	23.8 ± 2.3	25.1 ± 3.2	25.6 ± 4.3	0.198
A1c, %	4.9 ± 0.4	6.0 ± 0.2	7.5 ± 1.0	**0.000**
Presence of ApoE 4, %	6 (27.3%)	3 (17.6%)	2 (9.5%)	0.322
Duration of diabetes, years	NA	NA	8.1 ± 5.5	NA
Fasting blood glucose, mmol/L	4.88 ± 0.29	5.93 ± 0.77	7.88 ± 2.32	0.000
ICV, mm^3^	1,337,874 ± 123,899	1,321,660 ± 155,912	1,389,083 ± 112,327	0.244
GM, mm^3^	653,360 ± 55,447	627,307 ± 80,561	648,173 ± 45,923	0.392
WM, mm^3^	467,911 ± 47,462	445,708 ± 55,750	483,456 ± 44,627	0.070
CSF, mm^3^	216,603 ± 67,366	248,645 ± 63,028	257,453 ± 60,893	0.099

Note

Data are shown as mean ± *SD*. *N* (%): percentages are based on the individual categories.

The bold values represent *p* <0.05.

Abbreviations: A1c, plasma glycosylated hemoglobin; BMI, body mass index; CSF, cerebrospinal fluid; DBP, diastolic blood pressure; GM, gray matter; ICV, intracranial volume; SBP, systolic blood pressure; WM, white matter.

### 3.2 Blood glucose status and cognitive function

As shown in Table 2, no significant between‐group differences were identified for any of the cognitive tests. However, there were suggestive between‐group differences for executive function, as assessed by trail making test B (*p* = 0.073) and Stroop test C (*p* = 0.076). Then, multiple linear regression analysis was used to investigate the effect of A1c level on cognitive function. Increased A1c level was significantly associated with executive dysfunction, as assessed by trail making test B (*R* = 0.503, *p* = 0.0016) and Stroop test C (*R* = 0.506, *p* = 0.001).

**Table 2:** Between‐group differences in cognitive performance, as evaluated by analyses of covariance

	Nondiabetes *n* = 22	Prediabetes *n* = 17	Diabetes *n* = 21	*F*‐value	*p*‐Value
MoCa	25.1 ± 2.2	24.1 ± 2.9	23.4 ± 4.5	1.413	0.252
Verbal fluency test	45.9 ± 6.8	46.4 ± 8.2	44.4 ± 8.8	0.333	0.718
Immediate memory	36.4 ± 7.9	37.1 ± 7.8	37.9 ± 9.7	0.174	0.840
Delayed Memory	8.3 ± 2.4	7.1 ± 2.3	6.8 ± 2.5	2.480	0.085
Stroop test A	21.3 ± 12.7	20.1 ± 8.5	18.1 ± 6.8	0.600	0.552
Stroop test B	19.9 ± 6.6	21.8 ± 7.2	22.4 ± 11.2	0.474	0.625
Stroop test C	27.4 ± 7.1	34.7 ± 10.8	35.9 ± 11.8	2.702	0.076
TMT‐A	39.7 ± 12.6	41.5 ± 13.0	39.6 ± 14.1	0.118	0.889
TMT‐B	69.1 ± 19.4	80.2 ± 26.3	83.4 ± 28.1	2.726	0.073

Note

Data are mean ± *SD*.

Abbreviation: TMT, trail making test.

### 3.3 Blood glucose status and hippocampal subfields volume

**Figure 1:**
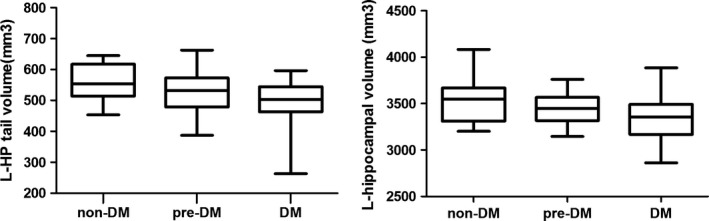
The between‐group difference of the left hippocampal volume and the left hippocampal tail volume. Compared with the nondiabetic group, the volume of the tail of hippocampus decreased significantly in both prediabetic and diabetic groups. L‐HP, left hippocampus

Table 3 shows the between‐group differences in hippocampal subfield volumes. Significant differences among groups were found in the total left hippocampal volume (*F* = 3.257, *p* = 0.046) and left hippocampal tail volume (*F* = 4.623, *p* = 0.014) after adjusting all the covariants (Figure 1). In addition, the post hoc Sidak testing showed that, compared with the nondiabetes group, the volumes of the left hippocampal tail were significantly reduced both in diabetes and prediabetes group. There was no interaction between A1c categories and age (all *p* > 0.05).

**Table 3:** Between‐group differences in hippocampal subfield volume (mm^3^), as evaluated by analyses of covariance

	Nondiabetes *n* = 22	Prediabetes *n* = 17	Diabetes *n* = 21	*F*‐value	*p*‐Value
Left hippocampus					
Total HPV	3,563.2 ± 318.6	3,456.9 ± 254.1	3,318.0 ± 354.0*	3.257	**0.046**
CA1	634.8 ± 65.4	616.8 ± 47.2	601.9 ± 72.2	1.442	0.245
CA2 + CA3	211.7 ± 27.1	207.1 ± 26.3	199.1 ± 24.0	1.306	0.279
CA4	263.8 ± 25.1	256.8 ± 24.2	249.9 ± 27.1	1.576	0.216
DG	307.9 ± 30.0	301.0 ± 25.6	289.6 ± 31.4	2.118	0.130
Hippocampal tail	561.3 ± 68.0	526.0 ± 72.5*	495.4 ± 74.1**	4.623	**0.014**
Fimbria	87.8 ± 15.4	88.5 ± 17.7	86.0 ± 22.2	0.094	0.910
Hippocampal fissure	163.0 ± 26.0	175.3 ± 32.2	161.9 ± 29.9	1.182	0.314
Molecular layer	583.1 ± 58.3	567.1 ± 39.4	545.0 ± 62.9	2.550	0.087
Subiculum	461.5 ± 45.7	445.7 ± 40.6	426.6 ± 60.6	2.609	0.082
Parasubiculum	63.8 ± 10.9	62.1 ± 11.9	64.6 ± 12.4	0.226	0.798
Presubiculum	328.6 ± 36.4	316.7 ± 31.4	306.2 ± 42.4	1.940	0.153
Right hippocampus					
Total HPV	3,747.1 ± 371.1	3,668.0 ± 264.2	3,519.6 ± 379.1	2.354	0.104
CA1	673.9 ± 83.9	668.1 ± 65.8	635.9 ± 73.6	1.526	0.226
CA2 + CA3	235.0 ± 32.3	231.3 ± 30.7	226.4 ± 32.0	0.392	0.677
CA4	283.8 ± 31.2	276.5 ± 29.8	266.8 ± 31.1	1.653	0.201
DG	329.7 ± 36.7	323.1 ± 32.1	308.9 ± 37.7	1.871	0.163
Hippocampal tail	600.2 ± 84.5	588.5 ± 66.4	560.3 ± 66.7	1.644	0.202
Fimbria	79.1 ± 17.6	87.2 ± 19.2	78.9 ± 27.5	0.846	0.434
Hippocampal fissure	178.6 ± 24.0	177.2 ± 36.1	177.5 ± 28.7	0.014	0.986
Molecular layer	617.4 ± 67.0	602.6 ± 44.8	575.6 ± 66.5	2.543	0.088
Subiculum	479.1 ± 48.1	458.7 ± 29.5	444.4 ± 54.9	1.493	0.233
Parasubiculum	59.0 ± 7.8	59.1 ± 10.1	58.8 ± 14.9	0.002	0.998
Presubiculum	328.1 ± 42.9	310.6 ± 46.9	303.4 ± 53.7	3.035	0.056

Note

Data are mean ± *SD*.

The bold values represent *p* < 0.05.

Abbreviations: CA, cornu ammonis; DG, dentate gyrus; HPV, hippocampal volume.

* Compared with nondiabetes group, *p* < 0.05.

** Compared with nondiabetes group, *p* < 0.01.

As presented in Table 4, among all participants, significant correlations were identified between the A1c level and the volume of left hippocampal tail (*R* = −0.334, *p* = 0.009), bilateral subiculum (*R*_Left_ = −0.291, *p* = 0.024; *R*_Right_ = −0.271, *p* = 0.036), and bilateral molecular layer (*R*_Left_ = −0.307, *p* = 0.017; *R*_Right_ = −0.283, *p* = 0.028) after adjusting for age, gender, body mass index, history of hypertension, level of cholesterol, duration of education, and the presence of ApoE4‐positive status.

**Table 4:** Partial correlation analysis: A1c with hippocampal subfields volume

	*R*‐value	*p*‐Value
Left hippocampus		
Total HPV	−0.314	**0.015**
CA1	−0.252	0.052
CA2 + CA3	−0.225	0.083
CA4	−0.237	0.068
DG	−0.288	**0.025**
Hippocampal tail	−0.334	**0.009**
Fimbria	−0.037	0.779
Hippocampal fissure	−0.030	0.821
Molecular layer	−0.307	**0.017**
Subiculum	−0.291	**0.024**
Parasubiculum	−0.021	0.871
Presubiculum	−0.219	0.092
Right hippocampus		
Total HPV	−0.263	**0.043**
CA1	−0.230	0.077
CA2 + CA3	−0.155	0.236
CA4	−0.245	0.059
DG	−0.258	**0.046**
Hippocampal tail	−0.213	0.102
Fimbria	−0.106	0.421
Hippocampal fissure	−0.051	0.696
Molecular layer	−0.283	**0.028**
Subiculum	−0.271	**0.036**
Parasubiculum	−0.091	0.491
Presubiculum	−0.298	**0.021**

Note

The bold values represent *p* <0.05.

Abbreviations: A1c, plasma glycosylated hemoglobin; CA, cornu ammonis; DG, dentate gyrus; HPV, hippocampal volume.

### 3.4 Volume comparison of bilateral hippocampal subfields

Table 5 presents the differences in volume between the left and right hippocampal subfields. The total and tail volumes of the left hippocampus were significantly smaller than the total and tail volumes of the right hippocampus, suggesting that the bilateral hippocampus is asymmetrical.

**Table 5:** Comparison of the volume between the left and right hippocampal subfields (mm^3^)

	Left hippocampus *n* = 60	Right hippocampus *n* = 60	*F*‐value	*p*‐Value
Total HPV	3,447.2 ± 327.3	3,645.1 ± 355.4	0.467	0.002
CA1	618.2 ± 64.0	659.0 ± 76.3	2.007	0.002
CA2 + CA3	206.0 ± 26.0	230.9 ± 31.4	1.927	0.000
CA4	257.0 ± 25.8	275.8 ± 31.1	1.474	0.000
DG	300.0 ± 30.0	320.5 ± 36.4	1.671	0.001
Hippocampal tail	531.1 ± 75.7	582.9 ± 74.5	0.052	0.000
Fimbria	87.4 ± 18.4	81.3 ± 21.9	0.682	0.101
Hippocampal fissure	166.1 ± 29.3	177.8 ± 28.9	0.965	0.029
Molecular layer	565.2 ± 56.9	598.6 ± 63.0	0.673	0.003
Subiculum	444.8 ± 51.6	461.1 ± 48.0	1.103	0.075
Parasubiculum	63.6 ± 11.6	59.0 ± 11.1	0.646	0.028
Presubiculum	317.4 ± 37.9	314.5 ± 48.4	1.131	0.717

Note

Data are mean ± *SD*.

Abbreviations: CA, cornu ammonis; DG, dentate gyrus; HPV, hippocampal volume.

## 4 DISCUSSION

In this cross‐sectional study, we used an automated volumetric segmentation method to accurately determine the hippocampal subfield volume in participants with and without diabetes, as well as those in the prediabetes stage. To the best of our knowledge, this is the first in vivo study to compare the hippocampal subfield volume differences in the early stage of diabetes. Our findings suggest that the left posterior hippocampus, which is mainly composed of the left hippocampal tail, was affected earlier in hyperglycemia‐associated hippocampal atrophy, even in prediabetic individuals. Furthermore, executive function may be the main cognitive domain damaged in individuals in the prediabetes stage.

It is well known that diabetes increases the risk of cognitive impairment in the elderly, in form of memory and executive dysfunction. However, similar to several previous studies (van den Berg et al., 2008; Luchsinger, Cabral, Eimicke, Manly, & Teresi, 2015), our data show that executive function is the main cognitive domain involved in the prediabetes stage. Traditionally, impairment in executive function has been attributed to cerebrovascular disease and disruption of frontal subcortical networks. In our study population, stroke events are excluded, and the degree of cerebral small vessel diseases is very mild (assessed by presence of lacuna and WMHs), which indicated that there may exist nonvascular pathological damage underlying the executive dysfunction in prediabetes stage. In fact, previous studies have shown that reduced hippocampal volume correlates with executive dysfunction, but not memory function in major depression (Frodl et al., 2006).

Similar to the findings of a previous report (Zhang et al., 2015), the present study also found that the volumes of the bilateral hippocampus, bilateral hippocampal molecular layer, and left DG were significantly reduced in participants with diabetes. These results strengthened the evidence that there is preferential involvement of certain hippocampal subfields in patients with diabetes. As the bilateral hippocampal volume was asymmetrical with differential function (Woolard & Heckers, 2012), it is better to separate the bilateral hippocampal subfields than to combine them when comparing among various groups (Zhang et al., 2015). We further sorted out prediabetes participants and found that the left hippocampal tail was the major hippocampal substructure affected in this early stage. Our results suggest that the volume loss in the left hippocampal tail may be an early biomarker for hyperglycemia‐associated hippocampal atrophy.

Hippocampus can be segmented anatomically and functionally into distinct subfields (head, body, and tail) along its ventrodorsal axis (Fanselow & Dong, 2010). In humans, functional connectivity in the hippocampal tail correlated positively with the thalamus and posterior cingulate cortex and promoted the formation of hippocampus‐associated cognitive function (Zarei et al., 2013). At present, the mechanisms underlying the hippocampal tail shrinkage vulnerability to hyperglycemia remain unclear, in which we speculate that hippocampal microangiopathy may play an important role. The arterial supply of the hippocampal tail originates from the P3 segment of the posterior cerebral artery, a peripheral artery that is often involved in diabetes (Umemura, Kawamura, & Hotta, 2017). Indeed, a prior clinical study showed that hyperglycemia can lead to small vascular and microvascular lesions in multiple brain regions, including the hippocampus (Sanahuja et al., 2016), while an experimental study using an animal model of diabetes demonstrated that antidiabetic drugs were able to partially restore abnormal amyloid‐beta transport across the blood–brain barrier and improve memory function (Chen et al., 2016). Other studies have shown that the hippocampal tail volume of patients with major depression was significantly smaller than that of the controls (Maller et al., 2012) and that diabetes was one of the most important risk factors for senile depression (Semenkovich, Brown, Svrakic, & Lustman, 2015). Therefore, structural and functional impairment of the hippocampal tail may be a common pathological manifestation of depression and diabetic brain damage.

There are several limitations in our study. First, the sample size of our study was relatively small, which may impede its generalizability. Second, we did not evaluate the microvascular complications such as microvascular lesions in retina and kidney, but we examined two main presentations of cerebral small vessel diseases (lacunae and WMHs). Third, random blood glucose level was not measured immediately before the neuropsychological assessment, which may affect the instant cognitive results. Nevertheless, this study clearly supports the view that for individuals with diabetes, the left posterior hippocampus, especially the hippocampal tail, may be affected earlier and associated with executive dysfunction. Additional studies, especially longitudinal studies, are needed to demonstrate the exact role of the hippocampal tail in diabetes‐associated cognitive impairment. Furthermore, clinicians should pay particular attention to adults in the prediabetes stage in order to prevent later cognitive impairment.

## CONFLICT OF INTERESTS

The authors declare that they have no competing interests and are alone responsible for the content and writing of the paper.

## AUTHORS' CONTRIBUTIONS

Sun Dong is a research associate of the conducted study, conceived and designed the study, analyzed and interpreted the data, and prepared the manuscript. Lu Dongwei conceived and designed the study, involved in neurologic examination, and prepared and wrote the manuscript. Junjian Zhang involved in substantial contribution to conception and study design, detected cognitive scale, and prepared and reviewed the manuscript. Jinyu Liang detected cognitive scale and prepared and reviewed the manuscript. Zhenmeng Sun and Jian Fang involved in MR scanning and postprocessing of MRI.

## ETHICS APPROVAL AND CONSENT TO PARTICIPATE

The study was approved by the medical ethics committee of Zhongnan Hospital of Wuhan University. Written informed consent was obtained from all participants. This study is registered at http://www.chictr.org.cn/index.aspx. The registration number is ChiCTR‐RNC‐12002205.

## CONSENT FOR PUBLICATION

Not Applicable.

## Data Availability

The datasets used in the current study available from the corresponding author on reasonable request.
